# Supplementary material for: Myeloid-derived suppressor cells exacerbate poly(I:C)-induced lung inflammation in mice with renal injury and older mice
Source: Front Immunol. 2023 Sep 25;14:1243851. doi: 10.3389/fimmu.2023.1243851 (PMC10560716; doi:10.3389/fimmu.2023.1243851)
Supplement: Supplementary file 1 [file DataSheet_1.docx]

Supplementary Material

# Supplemental Methods:

## MDSC differentiation *in vitro*

The *in vitro* differentiation of bone marrow (BM) cells into myeloid-derived suppressor cells (MDSCs; *in vitro* MDSCs) was performed as described previously (1, 2). Briefly, BM cells from C57BL/6J mice were stimulated with 40 ng/mL recombinant granulocyte-macrophage CSF (GM-CSF) (Peprotech, NJ, USA) for 4 days.

## *In vitro* suppression assay

CD8^+^ T cells were isolated from the spleens of C57BL/6J mice using the MojoSort magnetic cell separation system, as described previously (1, 2), and labeled with eFluor 670 proliferation dye (eBioscience, Thermo Fisher Scientific, CA, USA). eFluor 670-labeled CD8^+^ T cells were incubated with *in vitro* differentiated MDSCs at different ratios in a 96-well plate cultured with anti-mouse CD3ɛ antibody/anti-mouse CD28 antibody (BioLegend). After three days of incubation at 37 °C in 5% CO_2_, the proliferation of CD8^+^ T cells, as determined using the eFluor 670 fluorescence intensity, was analyzed using flow cytometry.

**Table S1.** List of primer sequences used in qRT-PCR analysis

| **Gene** | **Forward (5ʹ to 3ʹ)** | **Reverse (5ʹ to 3ʹ)** |
| --- | --- | --- |
| *Gapdh* | TGACCTCAACTACATGGTCTACA | CCGTGAGTGGAGTCATACTGG |
| *Arg1* | CCTATGTGTCATTTGGGTGGATG | GGTTGTCAGGGGAGTGTTGAT |
| *Nos2* | GGAGTGACGGCAAACATGACT | TAGCCAGCGTACCGGATGA |
| *Cybb* | CCTCTACCAAAACCATTCGGAG | CTGTCCACGTACCGGATGA |

qRT-PCR, quantitative reverse transcription-polymerase chain reaction; Genes: *Gapdh*, glyceraldehyde 3-phosphate dehydrogenase; *Arg1*, arginase 1; *Nos2*, nitic oxide synthase 2; *Cybb*, cytochrome b-245 beta polypeptide


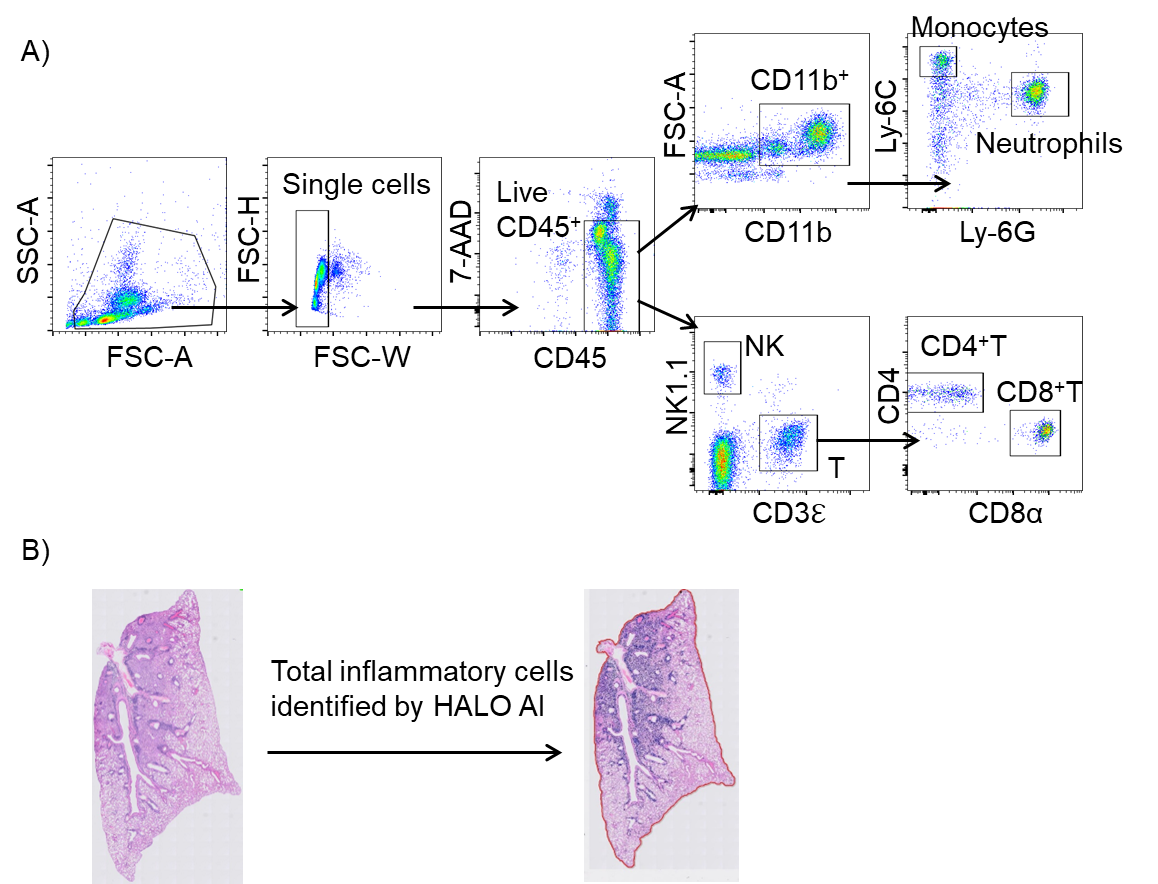


**Supplementary Figure 1.** (A) Gating strategy used for flow cytometric analysis. Monocytes (7AAD^−^CD45^+^CD11b^+^Ly-6G^−^Ly-6C^hi^), neutrophils (7AAD^−^CD45^+^CD11b^+^Ly-6G^+^Ly-6 C^int^), CD4^+^ T cells (7AAD^−^CD45^+^CD3ℇ ^+^CD4^+^NK1.1^−^), CD8^+^ T cells (7AAD^−^CD45^+^CD3ℇ^+^CD8α^+^NK1.1^−^), and NK cells (7AAD^−^CD45^+^CD3ℇ^-^NK1.1^+^). B) Representative image showing total inflammatory cells in heamtoxylin and eosin-stained sections identified using HALO AI.


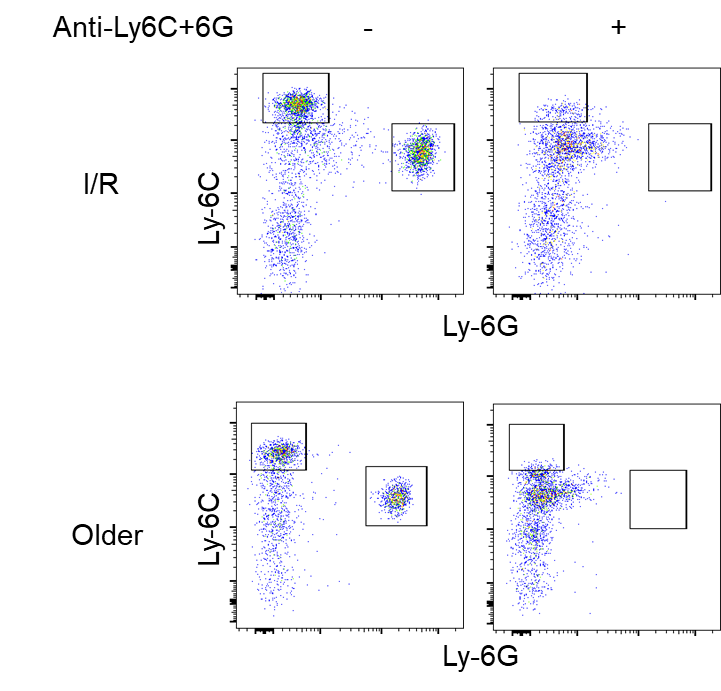


**Supplementary Figure 2.** Representative flow cytometry analyses of the frequencies of M-MDSCs (CD11b^+^Ly-6C^hi^Ly-6G^−^) and PMN-MDSCs (CD11b^+^Ly-6 C^int^Ly-6G^+^) in blood from phosphate-buffered saline (PBS)- or anti-Ly-6C+anti-Ly-6G antibody-treated mice with ischemia/reperfusion (I/R) injury or older mice with poly(I:C) challenge prior to sacrifice.

**
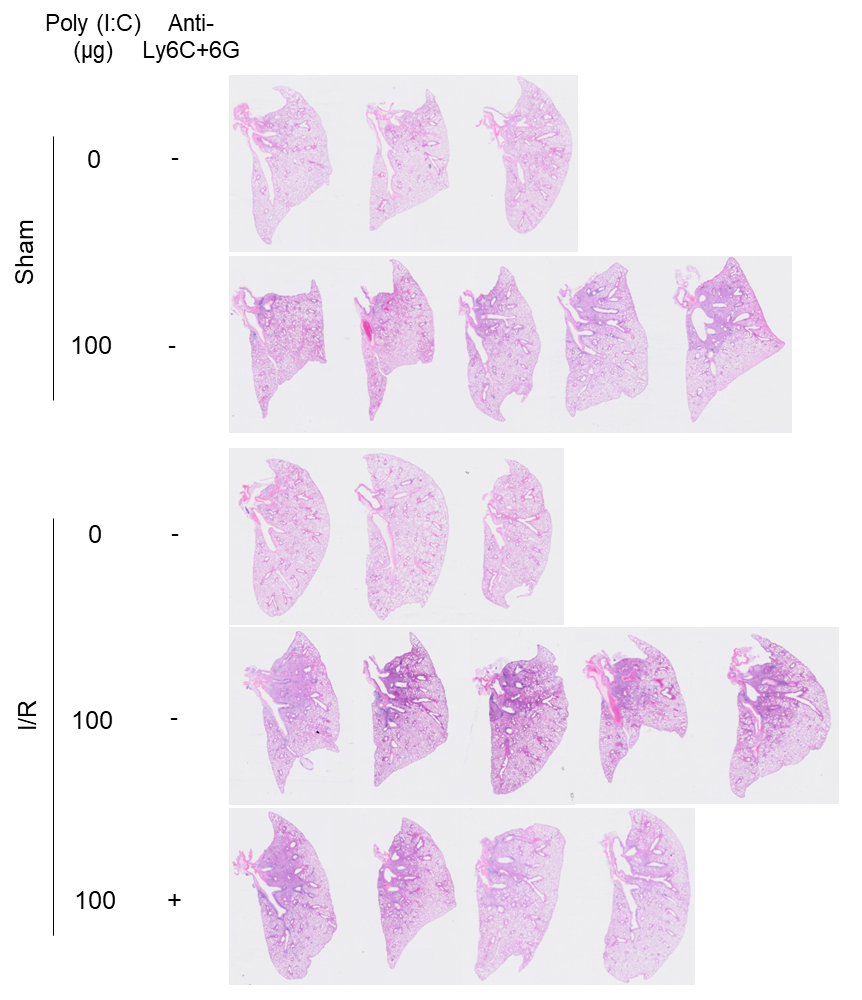
**

**Supplementary Figure 3.** Hematoxylin and eosin-stained lung sections from Sham, ischemia/reperfusion (I/R), and myeloid-derived suppressor cell (MDSC)-depleted I/R groups.


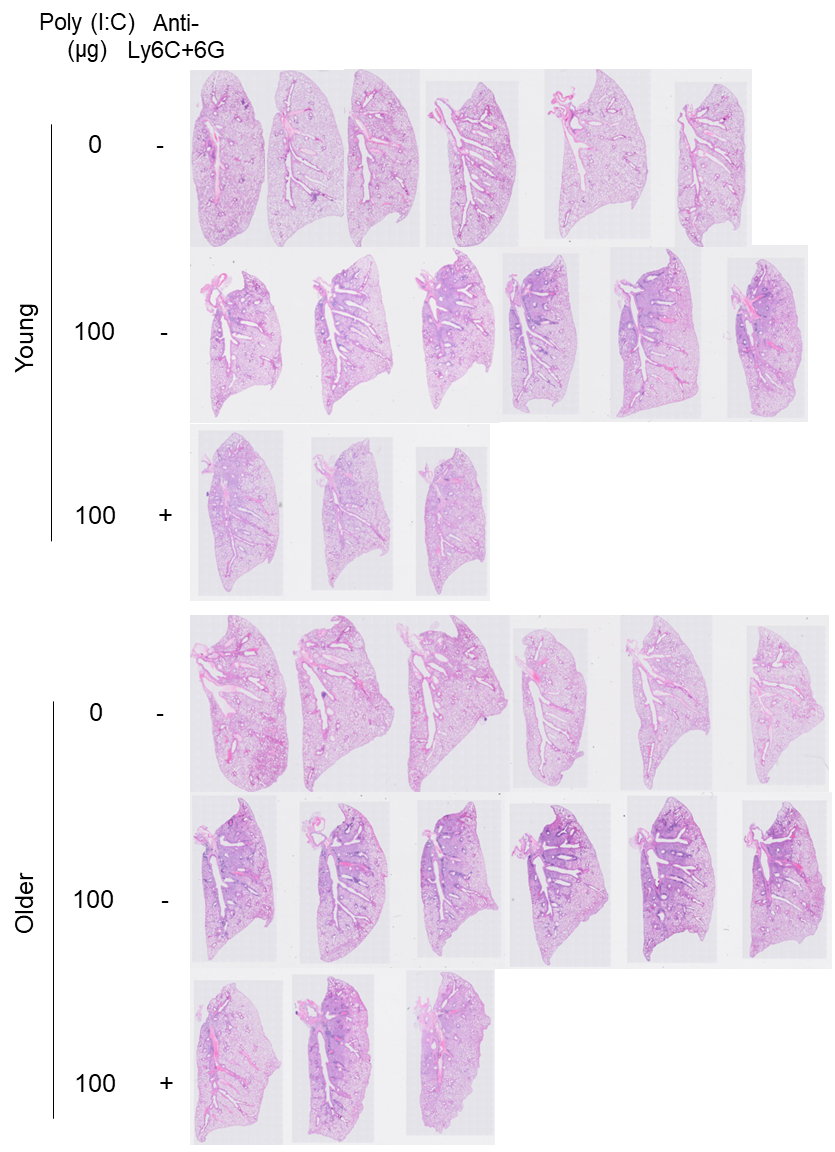


**Supplementary Figure 4.** Hematoxylin and eosin-stained lung sections from young, older, and myeloid-derived suppressor cell (MDSC)-depleted groups.

**
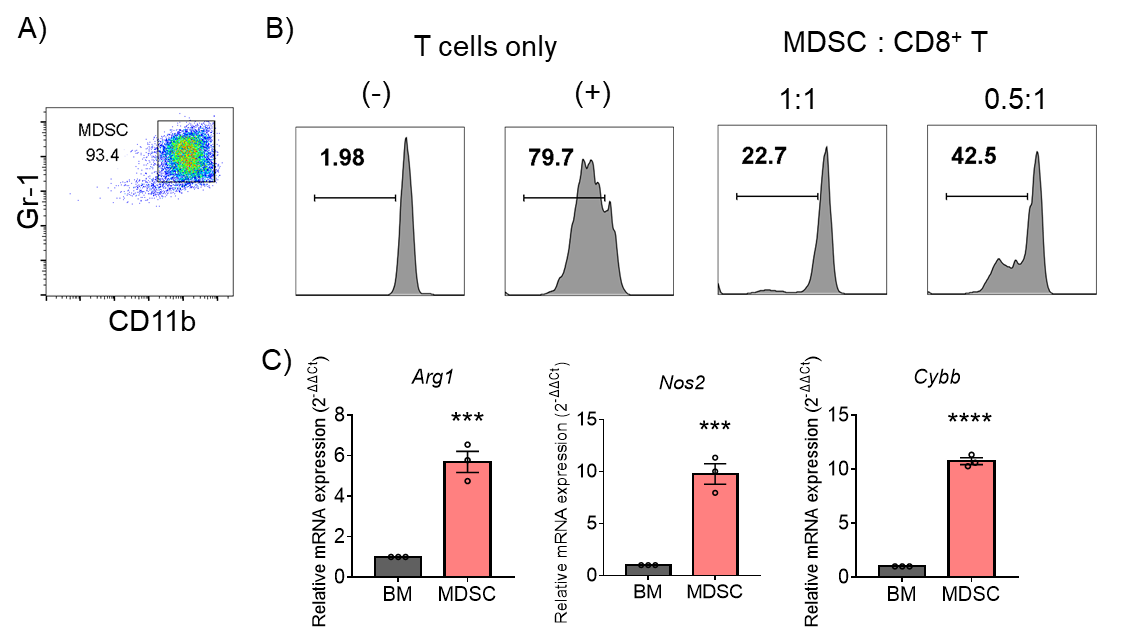
**

**Supplementary Figure 5.** (A) Flow cytometry analysis to determine the percentage of CD11b^+^Gr-1^+^ populations after four days of culture in medium supplemented with GM-CSF (40 ng/mL). (B) Histograms of eFluor 670 expression in CD8^+^ T cells. Myeloid-derived suppressor cells (MDSCs) were combined at a 1:1 or 0.5:1 ratio with eFluor 670-labeled CD8^+^ T cells, followed by stimulation with anti-CD3ε and anti-CD28 antibodies. (-) Mean CD8^+^ T cells without stimulation with anti-CD3ε Ab and anti-CD28 antibodies. (C) mRNA expression of *Arg1*, *Nos2*, and *Cybb* in bone marrow (BM) cells or MDSCs (CD11b^+^Gr-1^+^) measured using qRT-PCR (mean ± SEM; *n* = 3 per group. Student’s *t*-test: **p* < 0.05)


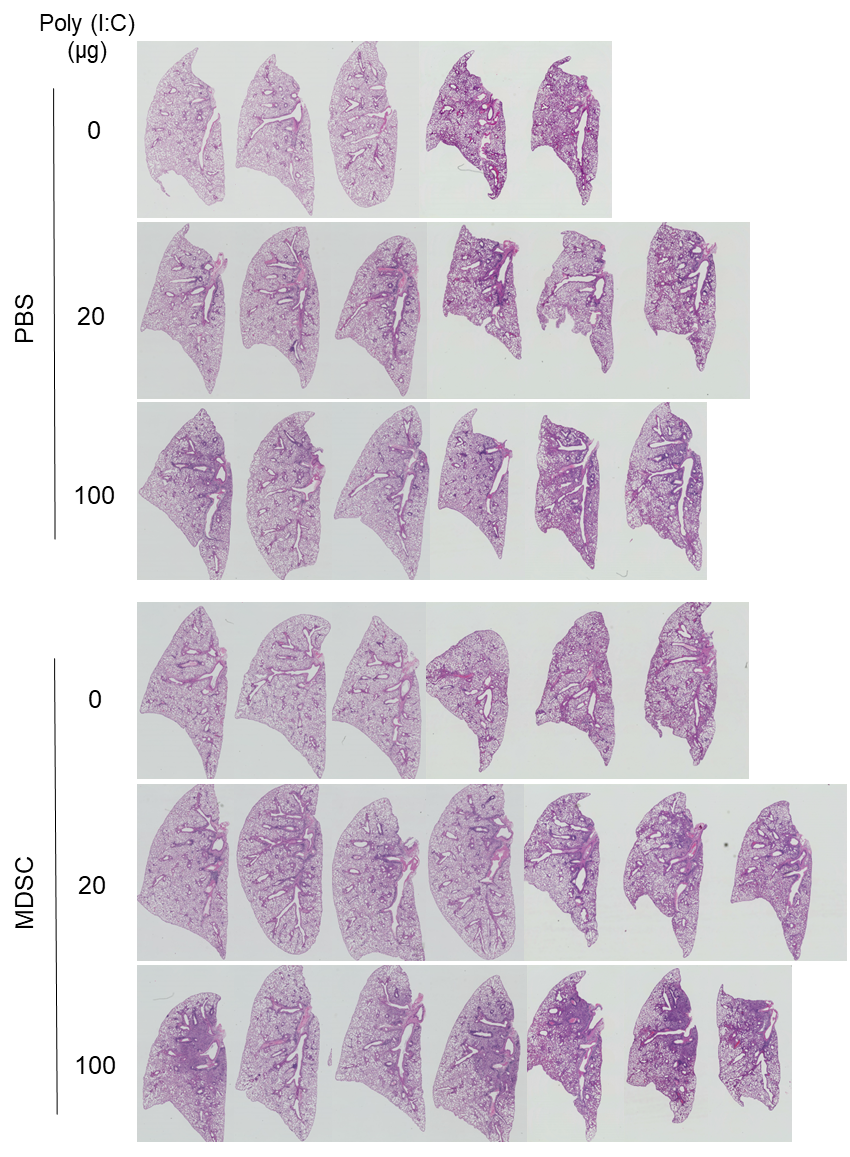


**Supplementary Figure 6.** Hematoxylin and eosin-stained lung sections from phosphate-buffered saline (PBS) or myeloid-derived suppressor cell (MDSC)-transferred mice.

**
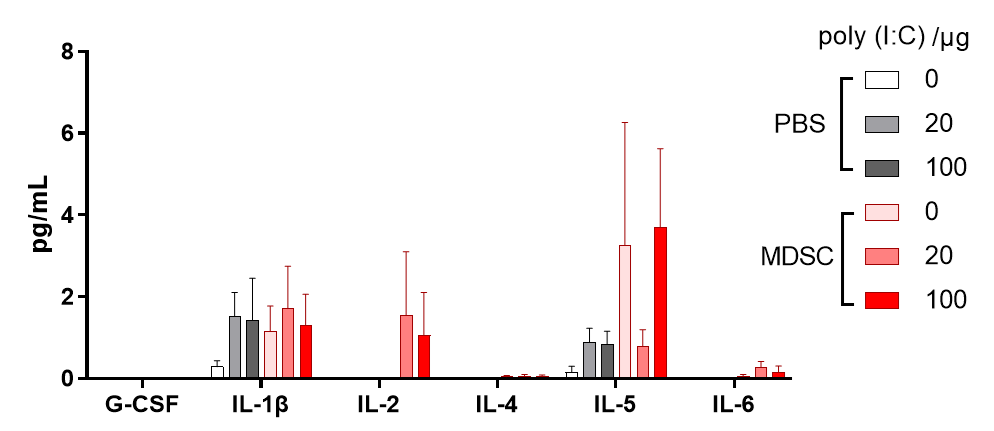
**

**Supplementary Figure 7.** Cytokines from brochoalveolar lavage fluid from phosphate-buffered saline (PBS)- or myeloid-derived suppressor cell (MDSC)-transferred mice were analyzed using Bio-Plex.

**References**

1. Xie Z, Kawasaki T, Zhou H, Okuzaki D, Okada N, Tachibana M. Targeting GGT1 eliminates the tumor-promoting effect and enhanced immunosuppressive function of myeloid-derived suppressor cells caused by G-CSF. Front Pharmacol (2022) 13**:**873792.

2. Xie Z, Ikegami T, Ago Y, Okada N, Tachibana M**.** Valproic acid attenuates CCR2-dependent tumor infiltration of monocytic myeloid-derived suppressor cells, limiting tumor progression. Oncoimmunology (2020) 9**:**1734268.
